# Pre-operative and early postoperative health-related quality of life of breast cancer woman: a Lebanese prospective study

**DOI:** 10.1186/s12905-023-02348-6

**Published:** 2023-04-20

**Authors:** Rana El Haidari, Virginie Nerich, Linda Abou-Abbas, Fadi Abdel-Sater, Amelie Anota

**Affiliations:** 1 Environments and Health Doctoral School, University of Bourgogne Franche-Comté, Besançon, France; 2 INSPECT-LB (Institut National de Santé Publique, Epidémiologie Clinique Et Toxicologie-Liban), Beirut, Lebanon; 3 INSERM UMR1098, University of Bourgogne, Franche-Comté, Besançon, France; 4 Department of Pharmacy, University Hospital of Besançon, Besançon, France; 5 Neuroscience Research Center, Faculty of Medical Sciences, Lebanese University, Beirut, Lebanon; 6 Laboratory of Molecular Biology and Cancer Immunology, Faculty of Science, Lebanese University, Hadath, Lebanon; 7 Direction of Clinical Research and Innovation & Human and Social Sciences Department, Centre Léon Bérard, Lyon, France; 8 French National Platform Quality of Life and Cancer, Besançon, France

**Keywords:** Breast Cancer, Cohort, Health-related quality of life, Lebanese women, Prospective

## Abstract

Breast cancer (BC) is a major health concern in Lebanon, with an increasing incidence rate due to advancements in treatment modalities. Evaluating the impact of the BC and its treatment on a woman's Health-Related Quality of Life (HRQoL), and comparing these patterns before and after breast conserving surgery is important to identify areas where interventions may be needed to improve the overall well-being of women with BC**.** This study aimed to evaluate the HRQoL pre and post-operative breast conserving surgery and just prior to initiation of adjuvant therapy in newly diagnosed patients with BC in Lebanon, specifically focusing on changes in body image. A prospective cohort study was conducted on 120 patients in two health care facilities in Lebanon, collecting sociodemographic and clinical data, and using the EORTC QLQ-C30 and QLQ-BR23 questionnaires to evaluate HRQoL. The outcomes were measured at baseline and then one-day post-operative breast surgery. Results revealed a statistically and clinically significant decrease in body image (mean difference of 8.1 points (95% 4.3;11.1)), physical functioning (mean difference of 6.1 points (95% 3.3;8.5)), and emotional functioning (mean difference of -8.4 points (95%-12.4; -4.9) after surgery. Positive change of physical functioning score was observed among married women. Positive change of emotional functioning score was observed among patients with poor body image score and high future perspective score. Our findings provide valuable insights for clinicians and researchers on the impact of breast conserving surgery on HRQoL in Lebanese women.

## Introduction

Breast cancer (BC) remains the most commonly diagnosed type of cancer among women worldwide. In Lebanon, BC incidence rates are continuously on the rise, with reported cases increasing from 1,451 in 2005 to 3,219 in 2018 [1, 2]. Moreover, approximately 920 (10%) BC-related deaths occurred in 2018 [3]. Despite this, advancements in early diagnosis and treatment methods have improved the chances of long-term disease-free survival for patients [4]. In recent decades, breast-conserving surgery (BCS) has been introduced as a modality to preserve the breast while maintaining survival rates for early stages of BC [5]. BCS is defined as “a combination of conservative surgery for resection of the primary tumor with or without surgical staging of the axilla” [5]. In the 1980s, randomized clinical trials showed that BCS followed by radiotherapy in women with early BC stage had excellent outcomes and optimal tolerability [6–8]. As a result, BCS has become a preferred alternative to radical mastectomy for treatment of early stage BC. A retrospective study conducted by El Saghir et al. in Lebanon reported that BCS rates in patients with early stage BC have increased from 48% between 1997–2002 to 64% between 2002–2010, while total mastectomy rates have decreased from 51 to 35% [9].

As a result of breast surgery, women experience considerable changes in their physical appearance, such as deformities in one or both breasts, surgery scars, and alopecia [10]. The breast is perceived as a major part of a woman's identity, a symbol of femininity, and women's capacity for rearing children and motherhood [11]. Therefore, any related deformities among breast cancer (BC) patients following surgery are deeply associated with poor body image perceptions [12, 13], which often leads to poor health-related quality of life (HRQoL) [14]. A recent study by Han et al. reported that poor body image perceptions in patients undergoing breast surgery have the potential to negatively impact their physical and psychological functioning, which can lead to a dramatic decrease in their HRQoL [15].

HRQoL of BC patients after BCS can also be affected by fatigue [16], which is a subjective feeling of tiredness, weakness, and lack of energy [17]. The impact of body image and fatigue on HRQoL can be extensive, reducing the patient's engagement in work as well as in personal and social activity [18, 19]. With the intention of supporting patients who adapt to their illness and report positive mental health states, several studies have been performed to identify psychological resources that predict better outcomes [9, 20–23]. One of the resources associated with HRQoL is habitual or dispositional optimism [24–26], a personality trait that describes the degree to which a person generally expects positive outcomes [27, 28]. Additionally, Patient-reported outcomes (PRO) instruments are used to assess symptoms and HRQoL from the patient's perspective [29].

Previous research on HRQoL in BC patients has been primarily conducted in Western countries, which may not be generalizable to the Middle Eastern population due to cultural and clinical differences. In the Middle East, breast cancer patients tend to be younger and are often diagnosed at an advanced stage, where BC is generally aggressive and requires several choices of treatment such as surgery [30]**.** There is a lack of information on the impact of active treatment on the HRQoL of BC patient in the Middle East, partcularly patients who have undergone BCS. Identifying risk factors that contribute to poorer HRQoL in Middle Eastern breast cancer patients would enable targeted interventions to improve their quality of life.

Thus, the main objective of the current study was to describe changes in the HRQoL according to body image pre- and post-operative BCS and just prior to initiation of adjuvant therapy in newly diagnosed patients with BC in Lebanon. Secondary objectives were 1) to assess changes in other HRQoL dimensions as well as in fatigue and optimism and pessimism between pre and postoperative breast surgery and 2) to identify sociodemographic and clinical factors associated with changes in the domains of HRQoL.

## Methods

### Study design and patients

A prospective study was conducted between January 2018 to March 2020 at two Lebanese hospitals, the Rafic Hariri University Hospital and the Sahel General Hospital. The inclusion criteria for the study were: female BC patients aged 18 or older who had recently been diagnosed with invasive early-stage breast cancer (stage I, IIa, and IIb), were scheduled to undergo breast surgery as primary treatment, were able to communicate in Arabic, had no history of other cancers or metastasis, and were not currently experiencing any acute illness that would affect their psychological well-being. Patients were excluded from the study if they had already received neo-adjuvant chemotherapy, were pregnant or breastfeeding, had an active infection, or had any underlying serious condition that would prevent them from receiving surgery. The study protocol was approved by the Institutional Review Board from the Rafic Hariri University Hospital in Beirut (IRB No. 18.007-Trans-CMO-(OM)) and the research ethics committee of the Sahel General Hospital. Written informed consent was obtained from all participants.

### Questionnaire

The questionnaire used was divided into five section: 1) Demographic characteristics, 2) Clinical characteristics, 3) the European Organization for Research and Treatment of Cancer (EORTC) QLQ-C30 cancer-specific questionnaire, 4) the EORTC QLQ-BR23 breast cancer specific module, 5) the Multidimensional Fatigue Inventory (MFI-20), and 6) The Life Orientation Test (LOT).

Sociodemographic characteristics including age, marital status, employment, education, and nationality were collected at baseline as well as the clinical variables including body mass index, time since diagnosis, family history of BC, menopausal status, comorbidities and cancer stage.

The EORTC QLQ-C30 cancer-specific questionnaire [31]includes 30-items that measure five functional scales (physical, role, emotional, cognitive and social functioning), global health status (GHS), financial difficulties and eight symptom scales (fatigue, nausea and vomiting, pain, dyspnoea, insomnia, appetite loss, constipation and diarrhoea). HRQoL scores were generated according to the EORTC scoring manual [32]. One score is generated per dimension and standardized on a 0 to 100 scale in order that a high score reflects a high GHS level, a high functional level and a high symptomatic level.

The EORTC QLQ-BR23 breast cancer specific module addresses specific issues of BC and should be used in conjunction to the QLQ-C30 [33]. It includes 23 items allowing to assess four functional scales (body image, sexual functioning, sexual enjoyment, future perspectives) and four symptom scales (systemic therapy side effects, breast symptoms, arm symptoms, upset by hair loss). As for the QLQ-C30, one score is generated per dimension on a 0–100 scale in order that a high score represents a high level of functioning and a high symptomatic level. The Arabic versions of the EORTC QLQ-C30 and QLQ-BR23 were found to be reliable and valid tools for assessment of quality of life in Arab patients with cancer [34].

The MFI-20 is a 20-item scale designed to evaluate during the four past week five dimensions of fatigue: general fatigue, physical fatigue, reduced motivation, reduced activity, and mental fatigue [35]. Subscores range from 4–20 scale with high score reflecting a high level of fatigue [36]. In this study, the Cronbach’s alpha value was 0.86.

The LOT is a 10-item self-report measure that assesses optimism and pessimism. The respondents indicates the extent to which they agreed with each of the items on a 5-point Likert scale from 0 (strongly disagree) to 4 (strongly agree). A total score ranging from 10 to 50 is calculated with higher scores indicating greater optimism and lower scores indicating lower optimism, often referred to as pessimism [27]. In our study, the Cronbach’s alpha value was 0.68.

The MFI-20 and The LOT were translated to the Arabic language and then back translated by two experts including linguists. The original and back-translated English versions were compared, and some minor edits were made to ensure the accuracy of the translation.

Participants were asked to complete the four scales at baseline (T0) (admission day), and one day after the breast surgery (T1). The estimated time to complete the questionnaire is between 30 and 45 min.

### Sample size calculation

The sample size was calculated using the PASS sample size software [37]. In order to detect a mean difference of at least 5 points after surgery compared to baseline in the body image targeted dimension, with a standard deviation (SD) of 20 points, a total sample size of 120 patients is required to achieve a statistical power of 80% and a bilateral type I error rate of 5% (using a paired T-test). According to the International Agency for Research on Cancer, an estimated population of 3219 new breast cancer cases in Lebanon in 2018 could be observed [3].

### Statistical analysis

The data was analyzed using the Statistical Package for Social Sciences software SPSS version 26. Baseline sociodemographic and clinical characteristics of the patients as well as baseline PRO scores were described using mean and SD and median and range for continuous variables and frequencies with percentages for qualitative variables. For the primary objective, the analysis was conducted on all patients who completed both pre- and post-surgery questionnaires. For secondary objectives, the analysis was performed on all patients who completed at least the baseline questionnaire. Only the functional scales of the EORTC questionnaires were analysed, as patients were included prior to the initiation of adjuvant therapy and did not have symptoms related to treatment. Indeed, the sexual functioning and sexual enjoyment dimensions of the QLQ-BR23 were not analysed since they were not filled out by the patients due to cultural sensitivity. The PRO scores of the EORTC QLQ-C30, QLQ-BR23, MFI-20, and LOT questionnaires were analyzed according to the guidelines for each questionnaire [32, 36, 38]. The minimal clinically important difference (MCID) was set at 5 points for each score of the EORTC QLQ-C30 and QLQ-BR23 questionnaires [33], 2 points for each score on the MFI-20 questionnaire [39], and 3 points, which was estimated as half of the SD observed at baseline of the LOT global score [40], as there was no validated estimate of MCID for the LOT questionnaire. The results were interpreted in relation to the MCID and statistical significance, to assess their clinical relevance. Mean HRQoL scores at pre- and post-surgery, as well as the mean change between pre- and post-surgery, were reported for each PRO scale. Paired t-tests were used to compare mean scores between pre- and post-surgery, and 95% confidence intervals (CIs) were calculated.

Generalized estimation equation (GEE) models were performed, with the change in body image score (i.e. the difference between pre-surgery and post-surgery body image scores) as the dependent variable in the first analysis, and two selected scores with the most important statistically and clinically significant differences observed as the dependent variables in the other two analyses. Independent variables were age, body mass index, marital status, educational level, employment status, nationality, menopausal status, comorbidities, family history of breast cancer, time since diagnosis, and cancer stage. The GEE approach accounts for the correlation between the repeated measures within a person. The GEE parameter estimates were expressed as the coefficients (β) and the 95% confidence intervals (95% CIs). All tests were two-sided, and statistical significance was set at a p-value of less than 0.05.

## Results

### Socio-demographic and clinical characteristics of participants

Between January 2018 and March 2020, a total of 120 patients were enrolled and analyzed. The baseline socio-demographic and clinical characteristics of the patients are summarized in Table 1. The mean age was 54.7 (9.3) years. The majority of women were of Lebanese nationality (*n* = 93 (77%)), married (*n* = 91 (79%)), currently not employed (*n* = 93 (77%)), and had completed a high or university level of education (*n* = 60 (51%)). The median length of time since diagnosis was (34 days). Nearly 60% of patients were diagnosed with disease stage II (*n* = 68 (56%)).

**Table 1:** Socio-demographic and clinical characteristics of included breast cancer patients (*n* = 120)

Variables	n	%
**Age,** mean (SD) 54.7 (9.3)		
**Marital status**		
Married	91	79.1
Single	13	11.3
Divorced	7	6.1
Widow	4	3.5
Missing	5	
**Education level**		
Below Primary school	12	10.4
Primary school	44	37.9
High school	47	40.5
University degree and above	13	11.2
Missing	4	
**Employment (Yes)**	27	22.5
**Nationality**		
Lebanese	93	77.5
Syrian	18	15.0
Palestinian	9	7.5
**BMI** in kg/m2, mean (SD) 28.1 (3.5)		
**Time since diagnosis** in days, median (range) 34.0 (21—62)		
**Menopause (Yes)**	71	59.2
**Comorbidities (Yes)**	57	47.5
**Family history of BC (Yes)**	32	26.6
**Stage**		
I	52	43.3
II	68	56.7
**Tumor size**		
T1	42	37.2
T2	71	62.8
Missing	7	
**Lymph node status**		
N0	45	39.8
N1	68	60.2
Missing	7	

Abbreviations: *BC* Breast cancer, *BMI* Body mass index, *n* number, *%* Percentage, *SD* Standard deviation

### Changes of patient-reported outcomes data after breast surgery

In the EORTC scales, a statistically and clinically significant decrease one day after surgery compared to baseline was observed in physical functioning with a mean difference of 6.1 points (95% CI: 3.3, 8.5), emotional functioning with a mean difference of -8.4 points (95% CI: -12.4, -4.9), and body image with a mean difference of 8.1 (95% CI: 4.3, 11.1), and a *p*-value of < 0.0001 (Table 2).

**Table 2:** Patient-reported outcomes scores before and after breast surgery

Questionnaire	n	Admission day (T0) Mean (SD)	N	One day after breast surgery (T1) Mean (SD)	Mean Difference T0-T1	95% CI	*p*-value
**EORTC QLQ-C30**							
*Global health status*	120	69.7 (14.5)	116	66.1 (15.2)	3.6	-0.4;6.1	0.09
*Functional scales*							
Physical functioning	120	92.6 (12.0)	115	86.5 (14.6)	6.1	3.3; 8.5	< 0.0001
Role functioning	120	90.2 (15.6)	114	86.5 (16.7)	3.7	-0.5; 7.8	0.08
Emotional functioning	118	65.3 (26.1)	116	73.7 (19.7)	-8.4	-12.4; -4.9	< 0.0001
Cognitive functioning	119	82.7 (22.4)	118	83.3 (22.0)	-0.6	-4.1; 2.9	0.72
Social functioning	117	87.8 (18.6)	119	85.0 (19.7)	2.8	-1.6; 7.3	0.21
**QLQ-BR23**							
*Functional scales*							
Body image	120	89.2(17.7)	116	81.0 (21.6)	8.1	4.3; 11.1	< 0.0001
Future perspective	115	51.0 (20.3)	111	53.1 (24.9)	-2.1	-8.1; 5.6	0.71
**MFI-20**							
General fatigue	118	13.0 (2.3)	118	14.2 (2.4)	-1.3	-2.0; -0.7	< 0.0001
Physical fatigue	118	13.9 (3.0)	117	14.3 (2.4)	-0.4	-1.0; 0.2	0.21
Reduced activity	118	16.3 (2.9)	117	15.2 (3.3)	1.1	0.2; 1.8	0.01
Reduced Motivation	118	13.1 (2.8)	117	14.0 (2.8)	-1.0	-1.7; -0.1	0.01
Mental fatigue	118	15.5 (2.5)	117	14.8 (2.4)	0.6	0.1; 1.2	0.04
**LOT**							
*LOT global score*	116	36.9 (3.8)	118	37.1 (3.6)	-0.2	-1.3; 1.1	0.90

Abbreviations: *CI* Confidence interval, *EORTC QLQ-C30* European organization for research and treatment of cancer QLQ-C30 cancer-specific questionnaire, *QLQ-BR23* Breast cancer specific module, *LOT* Life orientation test, *MFI-20* Multidimensional fatigue inventory, *T0* Admission day, *T1* One day after breast surgery

In the MFI-20 questionnaire, significant deterioration was observed in general fatigue with a mean difference of -1.3 points (95% CI: -2.0, -0.7) and a *p*-value of < 0.0001, and reduced motivation with a mean difference of -1.0 points (95% CI: -1.7, -0.1) and a p-value of 0.01. A statistically significant improvement was observed in reduced activity with a mean difference of 1.1 (95% CI: 0.2, 1.8) and a *p*-value of 0.01, and mental fatigue with a mean difference of 0.6 (95% CI: 0.1, 1.2) and a *p*-value of 0.04. However, no clinically significant difference was observed in the MFI-20 scores with a mean difference lower than the MCID of 2 points (Table 2).

### Factors associated with the change in health related quality of life domains after breast surgery

Table 3 displays the generalized estimating equations of the factors associated with the change in body image score. Results showed that married women (*p* = 0.01), those with low body image scores before surgery (*p* < 0.0001), and those with high future perspective scores at baseline (*p* = 0.02) had a significant improvement in body image scores after surgery.(Table 3).

**Table 3:** Results of multivariate generalized estimating equation of factors associated with the change in body image after breast surgery ( *N* = 120)

Factors	References	Generalized Estimating Equations		
		beta	95%CI	*p*-value
Age*		0.32	-0.38;0.83	0.37
Marital status	Married	-9.43	-15.2; -1.02	0.01
Education level	Secondary school or higher	3.26	-4.62; 7.28	0.54
Employment	No	9.68	-19.2;1.49	0.48
Nationality	Lebanese	10.56	-2.6;4.27	0.41
BMI*		0.67	-0.18;1.53	0.12
Menopause	No	5.19	-9.33;19.7	0.25
Comorbidities	No	0.70	-6.64;8.05	0.85
Family history of BC	No	5.78	-4.03;15.6	0.24
Stage	I	-8.9	-19.77;5.92	0.13
Tumor size	T1	6.32	-10.86; 9.08	0.59
Lymph node status	N0	1.95	-5.79;8.21	0.73
Body image*		-0.38	-0.56;-0.19	< 0.001
Future perspective*		0.12	0.01;0.23	0.02
General fatigue*		1.34	-0.20;2.89	0.09
Physical fatigue*		0.82	-0.50;2.14	0.22
Reduced activity*		-1.96	-3.71;0.21	0.07
Reduced Motivation*		-0.91	-2.30;0.48	0.99
Mental fatigue*		-0.75	-2.14;0.63	0.28
LOT global score *		0.31	-0.31;0.94	0.32

Abbreviations: *CI* Confidence interval, *BC* Breast cancer, *beta* unstandardized regression coefficient, *BMI* Body mass index, *LOT* Life orientation test, *N* Number

^*^: continuous variables

The two selected scores for multivariable analysis with mostly important significant results were physical functioning and emotional functioning.

In the Table 4, it was found that married women (*p* = 0.03) had a significantly higher improvement in physical functioning score post-surgery compared to their counterparts. Additionally, patients with low physical functioning before surgery (*p* < 0.0001) had an improvement in physical functioning score after surgery (Table 4).

**Table 4:** Results of multivariate generalized estimating equation of factors associated with the change in physical functioning after breast surgery (*N* = 120)

Factors	References	Generalized Estimating Equations		
		**beta**	**95%CI**	***p*-value**
Age*		-0.33	-0.79;0.11	0.44
**Marital status**	Married	-8.55	-14.3;2.73	**< 0.001**
**Education level**	Secondary school or higher	-0.16	-7.29;6.97	0.96
**Employment**	No	1.81	-5.46′9.09	0.62
**Nationality**	Lebanese	3.84	-3.35;11.03	0.29
BMI*		-0.59	-1.13;-0.05	0.30
**Menopause**	No	-0.98	-10.19;8.23	0.83
**Comorbidities**	No	0.21	-3.45;3.88	0.90
**Family history of BC**	No	2.03	-3.29;7.36	0.45
**Stage**	I	7.14	-0.28;15.41	0.12
**Tumor size**	T1	10.3	5.60;13.20	0.44
**Lymph node status**	N0	-1.12	-6.25;3.99	0.66
Physical functioning*		-0.30	-0.54;-0.06	**0.01**
Body image score*		0.06	-0.07;0.21	0.36
Future perspective*		0.08	-0.00;0.16	0.06
General fatigue*		-0.52	-1.51;0.47	0.30
Physical fatigue*		0.39	-0.64;1.42	0.46
Reduced activity*		-0.75	-1.48;-0.02	0.05
Reduced Motivation*		-0.65	-1.90;0.59	0.30
Mental fatigue*		0.09	-1.15;1.35	0.87
**LOT global score** *		0.22	-0.13;0.58	0.22

Abbreviations: *CI* Confidence interval, *BC* Breast cancer, *beta* unstandardized regression coefficient, *BMI* Body mass index, *LOT* Life orientation test, *N* Number

^*^: continuous variables

In the Table 5, a significant improvement in emotional functioning scores was observed among patients with low emotional functioning scores before surgery (*p* < 0.0001) (Table 5).

**Table 5:** Results of multivariate generalized estimating equation of factors associated with the change in emotional functioning after breast surgery (*N* = 120)

Factors	References	Generalized Estimating Equations		
		**beta**	**95%CI**	***p*-value**
Age*		-0.38	-0.90;0.14	0.15
**Marital status**	Married	-11.09	-17.09;-5.08	**< 0.0001**
**Education level**	Secondary school or higher	-0.22	-9.64;9.01	0.96
**Employment**	No	0.92	-6.19;8.03	0.80
**Nationality**	Lebanese	0.77	-6.25;7.80	0.83
BMI*		-0.49	-1.15;0.16	0.13
**Menopause**	No	-0.77	-11.85;10.31	0.89
**Comorbidities**	No	1.62	-2.97;6.22	0.48
**Family history of BC**	No	1.93	-4.31;8.17	0.54
**Stage**	I	10.34	-19.82;0.85	0.63
**Tumor size**	T1	10.9	-0.23;11.65	0.54
**Lymph node status**	N0	-1.63	-7.18;3.91	0.56
Emotional functioning*		-0.04	-0.15; -0.60	**< 0.0001**
Body image score*		0.04	-0.08;0.18	0.48
Future perspective*		0.06	-0.04;0.16	0.27
General fatigue*		-0.16	-1.11;0.79	0.73
Physical fatigue*		0.32	-0.76;1.41	0.56
Reduced activity*		-0.62	-1.37;0.12	0.10
Reduced Motivation*		-1.01	-2.24;0.21	0.10
Mental fatigue*		0.18	-1.03;1.40	0.76
**LOT global score** *		0.38	-0.03;0.06	0.07

Abbreviations: *CI* Confidence interval, *BC* Breast cancer, *beta* unstandardized regression coefficient, *BMI* Body mass index, *LOT* Life orientation test, *N* Number

^*^: continuous variables

## Discussion

This study aimed to describe changes in the HRQoL in relation to body image before and after breast conserving surgery (BCS) among women newly diagnosed with breast cancer and to explore sociodemographic and clinical factors associated with changes in the domains of HRQoL. The study found that early breast cancer women's HRQoL change significantly after surgery in some domains, including physical function, emotional function and body image. The study also identified several risk factors for poor HRQoL, including decreased body image and decreased future perspective. The study found that married women, low body image and high future perspective score were significantly associated with improvement in body image score after surgery. In terms of physical functioning, married women, and low physical functioning before surgery were found to be significant predictors of improvement in physical functioning after surgery. For emotional functioning, low emotional functioning before surgery was found to be a significant predictor of improvement in emotional functioning after surgery.

Our study revealed that BC women had a statistically and clinically significant increase in HRQoL according to body image one day after surgery (mean difference of -0.38, *p* < 0.0001). However, in the GEE method, marriage was found to be the only independent predictor of the clinically relevant increase of postoperative body image score. This finding is contradictory with others studies conducted by Engel et al. [41] and Duggal et al. [42]; They found that women had lower body image scores after breast cancer surgery, suggesting that women did not like their appearance, did not feel whole, were unhappy with their breast and scar. However, body image changes must be wisely assessed to plan target intervention programs for improving body image, which would be beneficial for improving HRQoL of BC patients.

Our study also revealed a statistically significant postoperative increase in physical functioning. This results are inconsistent with similar investigations based on statistical significance showed in western countries [42–44]. A cohort of 87 Brazilian patients was conducted in 2017, Dell’Antônio Pereira et al. detected a statistically significant decrease in physical functioning (measured with EORTC QLQ-C30) 15 days after breast surgery [42]. Also, a prospective study conducted in Denemark by Andersen et al. on a sample of 278 BC women who underwent BCS, suggest that reduction of physical functioning after surgery was related to impairment of daily, work and social activities, as well as environmental factors and patient-related factors [44]. However, none of these studies analyzed their data using the MCID concept, which offers more clinical confidence to results in a setting with subjective measurements. Further research in this area is needed. However, healthcare professionals should encourage BC patients to be physically active as their abilities and conditions.

Furthermore, findings showed that patients had a statistically significant increase in HRQoL according to the emotional functioning subscale post-operatively as compared to the admission day. This is contradictory to findings from previously published western studies [13, 15, 21, 45]. However, in Arab countries, BC patients may show feelings of comfort and stability after completing breast surgery, as they believe that the malignant disease has been removed, which can support their hope for a cure [46]. Additionally, low body image score and low future perspective score before surgery were identified as the only independent predictors of the clinically relevant increase of post-operative emotional functioning subscale. One possible explanation for this could be that the fact that the cancer has been removed and the patient is able to start normal functioning again following the surgery. Another explanation may be attributed to the absence of education for health professionals about the differences between types of operations. Additionally, patients may expect more severe symptoms after the operation, but in fact, BCS does not typically cause severe side effects, which can contribute to an increase in post-operative emotional functioning. BC patients are particularly susceptible to emotional supportive therapy by healthcare professionals, which can help in the expression of emotions and teaching patients how to handle the problems associated with cancer. Once emotional needs have been identified, interventions should include: providing information to patients and families about their emotional state, explaining the best ways to share and express feelings within the family sphere, and teaching patients how to detect negative feelings and handle them through emotional self-control techniques.

Results showed a statistically significant deterioration in general fatigue and reduced motivation as measured by the MFI-20 questionnaire. This is in line with the findings of Rotonda et al. who suggest that patients with breast cancer who present with greater fatigue before surgery have a greater risk of experiencing postsurgical fatigue [47]. Additionally, a statistically significant improvement was observed in mental fatigue. This supports the observed improvement in emotional functioning post-operation. These findings may be attributed to social and familial support and communication with patients after surgery. However, it should be noted that none of these results are clinically significant.

This is the first prospective longitudinal study of HRQoL among breast cancer patients living in Lebanon. Previously validated measurement instruments for preoperative and postoperative analysis of HRQoL were used. The application of the MCID concept (instead of a statistical difference only) allowed to test associations with greater clinical relevance [48]. However, it is important to note that our study had a small sample size and a relatively short follow-up period This study was conducted on a sample of 120 women living in Lebanon, who undewent BCS to remove breast cancer in the first or second stage. It is a sample of convenience, not population based, generalizability of the results to the whole Lebanese population is not possible because data was collected from two hospitals that are reference centers in Beirut city for the treatment of BC. Additionally, the follow-up was set to one day after surgery, which has been applied in previous studies [45, 47]. However, this interval is considered insufficient as the effect of surgery on HRQoL usually occurs within the first few days and sometimes months after surgery [48, 49]. Follow-up questionnaires should be administered at multiple time points, including after postoperative irradiation, to capture the full spectrum of patients' experiences and changes in their quality of life. This will provide a more comprehensive understanding of the postoperative recovery and quality of life for BCS patients. We also acknowledge that further research is needed to fully understand the impact of different axillary surgical procedures and the breakdown of axillary surgery on QOL in breast cancer patients. The collection of these data was unfortunately not possible in this study.

Based on our findings, it is important for medical professionals to be involved with breast cancer patients before and after surgery in Lebanon in order to improve postoperative physical and emotional functions and body image. This includes providing education and information about the surgery and its potential impact on the patient's physical and emotional well-being, as well as offering support and resources for managing any physical or emotional challenges that may arise. Additionally, medical professionals should also be aware of cultural and societal factors that may influence the patient's experience and provide appropriate support and resources to address these factors. By providing comprehensive care and support before and after surgery, medical professionals can help breast cancer patients in Lebanon to better cope with the physical and emotional challenges of surgery and improve their overall quality of life.

## Conclusions

Our study offers insight for healthcare professionals and researchers by analyzing the impact of BCS on the quality of life of women with early stage breast cancer. Our findings indicate that surgery leads to significant changes in certain aspects of quality of life, such as physical function, emotional well-being, and body image. Additionally, certain risk factors for poor quality of life, such as negative body image and reduced outlook for the future, were identified. To further validate these results and devise interventions to enhance quality of life, future research should include a larger sample size and longer follow-up period.

## Data Availability

The data that support the findings of this study are available from the corresponding author upon reasonable request.
